# AI-enabled pipeline for virus detection, validation, and SNP discovery from next-generation sequencing data

**DOI:** 10.3389/fgene.2024.1492752

**Published:** 2024-11-11

**Authors:** Abozar Ghorbani, Mahsa Rostami, Pietro Hiram Guzzi

**Affiliations:** ^1^ Nuclear Agriculture Research School, Nuclear Science and Technology Research Institute (NSTRI), Karaj, Iran; ^2^ Department of Surgical and Medical Sciences, Magna Graecia University of Catanzaro, Catanzaro, Italy

**Keywords:** virus detection, next-generation sequencing, bioinformatics analysis, SNP discovery, viral genomics, AI-assisted genomics pandas

## Abstract

**Background and Aims:**

The rapid and accurate detection of viruses and the discovery of single nucleotide polymorphisms (SNPs) are critical for disease management and understanding viral evolution. This study presents a pipeline for virus detection, validation, and SNP discovery from next-generation sequencing (NGS) data. The pipeline processes raw sequencing data to identify viral sequences with high accuracy and sensitivity by integrating state-of-the-art bioinformatics tools with artificial intelligence.

**Methods:**

Before aligning the reads to the reference genomes, quality control measures, and adapter trimming are performed to ensure the integrity of the data. Unmapped reads are subjected to *de novo* assembly to reveal novel viral sequences and genetic elements.

**Results:**

The effectiveness of the pipeline is demonstrated by the identification of virus sequences, illustrating its potential for detecting known and emerging pathogens. SNP discovery is performed using a custom Python script that compares the entire population of sequenced viral reads to a reference genome. This approach provides a comprehensive overview of viral genetic diversity and identifies dominant variants and a spectrum of genetic variations.

**Conclusion:**

The robustness of the pipeline is confirmed by the recovery of complete viral sequences, which improves our understanding of viral genomics. This research aims to develop an auto-bioinformatics pipeline for novel viral sequence discovery, *in vitro* validation, and SNPs using the Python (AI) language to understand viral evolution. This study highlights the synergy between traditional bioinformatics techniques and modern approaches, providing a robust tool for analyzing viral genomes and contributing to the broader field of viral genomics.

## 1 Introduction

Viruses pose significant threats to human health, agriculture, and the environment, causing a wide range of infectious diseases in humans, animals, and plants. Rapid and accurate detection of viral pathogens is crucial for effective disease management, outbreak surveillance, and vaccine development. Traditional diagnostic methods for virus detection, such as polymerase chain reaction (PCR) and serological assays, have limitations in terms of sensitivity, specificity, and throughput (Cassedy et al., 2021).

Single Nucleotide Polymorphisms (SNPs) are the most common type of genetic variation in viruses, phages, and viroid playing a crucial role in disease susceptibility, drug response, and evolutionary adaptation (Zamai, 2020). However, the identification and characterization of SNPs from genetic data is challenging due to sequencing errors, alignment ambiguities, and genomic complexity. Traditional SNP calling methods often rely on heuristic rules and statistical models that can lead to false-positive or false-negative results (Nogales and L. DeDiego, 2019).

In recent years, the advent of Next-Generation Sequencing (NGS) technologies has revolutionized the field of genomics, enabling researchers to obtain vast amounts of genetic data with unprecedented speed and efficiency. NGS-based approaches offer a promising alternative as they enable comprehensive and unbiased characterization of viral communities in diverse biological samples. This flood of data has opened new avenues for studying the genetic makeup of various organisms, including viruses, and facilitated the identification of genetic variations such as SNPs (Wang et al., 2022). Furthermore, the integration of Artificial Intelligence (AI) tools and algorithms into bioinformatics pipelines has improved our ability to analyze and interpret complex genomic datasets, which has led to significant advances in virus detection, validation and SNP discovery (Lin et al., 2023).

In the realm of personalized medicine, pipelines can play a pivotal role in identifying viral pathogens and genetic variations that influence disease susceptibility and drug response in individual patients. This capability has the potential to revolutionize treatment strategies by enabling more targeted and effective interventions (Albahri et al., 2020). Moreover, pipelines in agriculture can contribute to the rapid identification and characterization of viral pathogens infecting crops, facilitating timely disease management and crop protection measures (Cob-Parro et al., 2024). In addition, pipelines can help monitor and track viral outbreaks in wildlife populations as part of environmental monitoring, thus curbing the spread of infectious diseases in ecosystems (Tuia et al., 2022).

In this study, we present a comprehensive pipeline for virus detection, validation, and SNP discovery from NGS data. Our pipeline integrates state-of-the-art bioinformatics tools to process raw sequencing data, align it to reference genomes, and identify viral sequences and SNPs with high accuracy and sensitivity. We demonstrate the effectiveness of our approach through a series of case studies and validation experiments that highlight its potential applications in clinical diagnostics, epidemiological surveillance, and evolutionary studies.

## 2 Materials and methods

**FIGURE 1:**
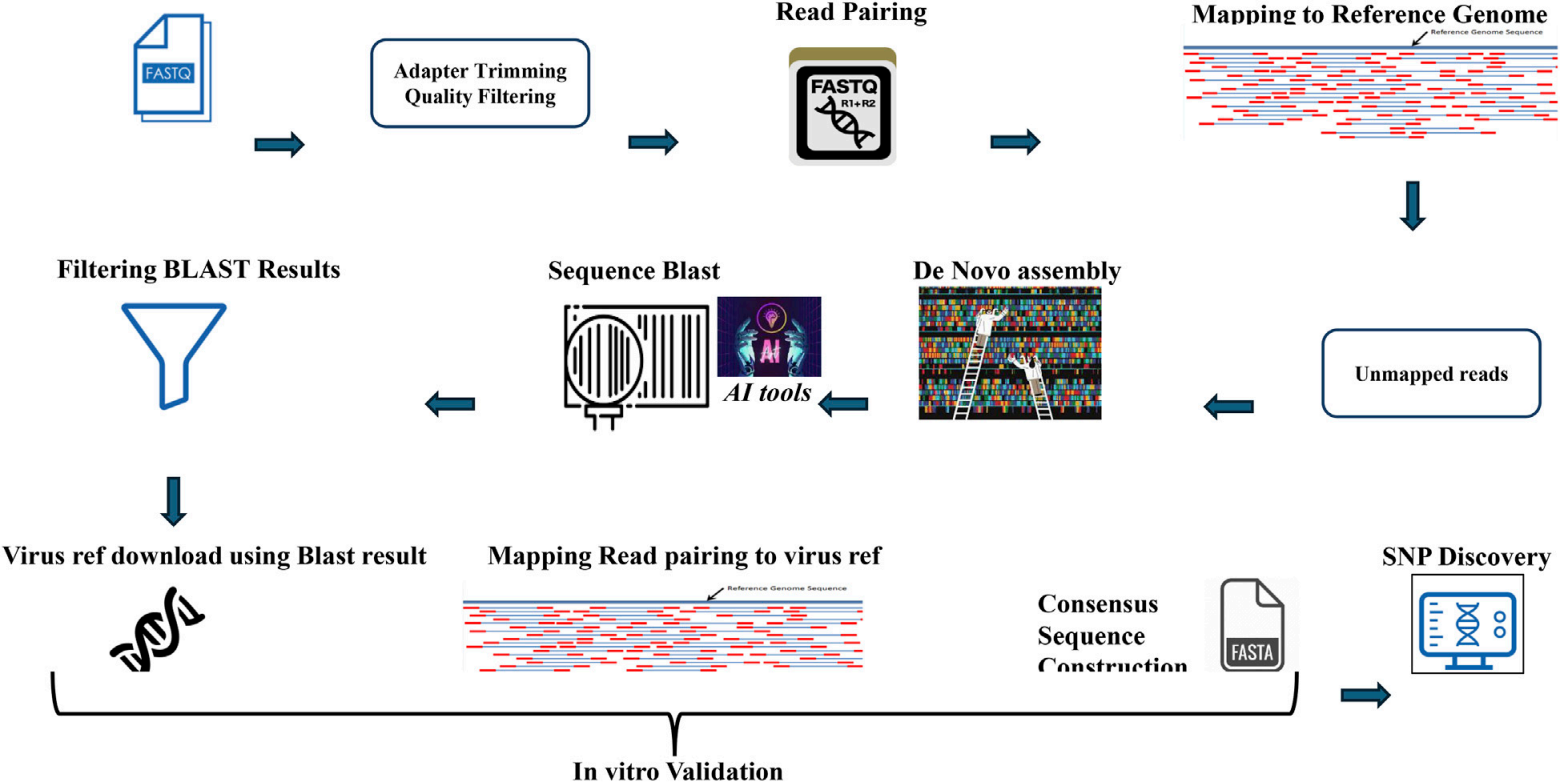
Viral sequence and SNP discovery: A python-powered odyssey. This Python-based workflow transforms raw NGS data into verified viral sequences. It includes quality filtering, read pairing, mapping, *de novo* assembly, and SNP discovery. *In vitro* validation ensures accuracy.

The methodology begins by explaining the range of software tools used in the comprehensive AI-powered pipeline for virus detection, validation, and SNP discovery from NGS data. The script uses a variety of software tools to perform different tasks. Here is a breakdown of the tools used and whether they need to be installed (Table 1). The script uses several software tools for data processing and analysis. Some tools, such as cutadapt, samtools, MegaHit, Biopython, and NCBIBlast+, must be installed separately. Built-in Python modules (os, random, subprocess) and functionalities within Biopython (Entrez, SeqIO) are readily available. Commonly used tools such as gzip and pandas may already be installed on the systems, but a check is recommended. The code is available in the Git Hub (https://github.com/Abozarghorbani/AI-Enabled-Virus-Detect). Figure 1 shows a comprehensive Python-based pipeline for converting raw NGS data into verified viral sequences (Figure 1).

**TABLE 1:** Software tools used in the Python script. This table summarizes the software tools used in the script, indicating whether they need separate installation or are readily available.

Tools	Version	Open source	Need to install	Description	URL
cutadapt	4.8	Yes	Requires Installation^c^	Trims adapter sequences from Illumina reads	https://pypi.org/project/cutadapt/
gzip	-	Likely Available^a^	Likely Available	Compresses and decompresses files	https://docs.python.org/3/library/gzip.html
samtools	1.12	Yes	Requires Installation	Manipulates alignments in SAM/BAM format	http://www.htslib.org/download/
subprocess	-	Pre-installed^b^	No	Python module to run external commands	https://docs.python.org/3/library/subprocess.html
os		Pre-installed	No	Python module for interacting with the operating system	https://docs.python.org/3/library/os.html
random	1	Pre-installed	No	Python module for generating random numbers	https://docs.python.org/3/library/random.html
MegaHit	v1.2.9	Yes	Requires Installation	Performs *de novo* assembly of sequencing reads	https://github.com/tonikelope/megabasterd
Biopython	1.83	Yes	Requires Installation	Collection of Python tools for biological analysis	https://biopython.org/
pandas	2.2.2	Yes	Requires Installation	Used for data manipulation and analysis	https://pandas.pydata.org/
NCBIBlastn/NCBIBlastx	BLASTn+ 2.15.0 /BLASTx+ 2.23.0	Yes	Requires Installation	Performs nucleotide or protein similarity searches	https://blast.ncbi.nlm.nih.gov/
Entrez	2.1.3	Likely Available	Likely Available	Part of Biopython for Entrez database access	https://www.ncbi.nlm.nih.gov/search/
SeqIO	1.83	Likely Available	Likely Available	Part of Biopython for sequence input/output	https://biopython.org/wiki/Documentation
pysam	0.22.1	Yes	Requires Installation	For manipulating alignments in SAM/BAM format	https://pysam.readthedocs.io/en/latest/
Counter	-	Pre-installed	No	Python module for creating collections of key-value pairs	-
Minimap2	2.28	Yes	Requires Installation	Performs alignment of sequencing reads	https://github.com/samtools/www.htslib.org

^a^ Likely Available: These tools are commonly included in scientific computing environments and might already be on your system. Check with your package manager to confirm availability.

^b^ Pre-installed: These modules come bundled with Python and don’t require separate installation.

^c^ Requires Installation: These tools need to be installed separately using their respective websites or package managers.

### 2.1 Trimming paired-end reads (data preparation, adapter trimming, and quality filtering)

The raw paired-end sequencing data obtained from NGS experiments were stored in compressed FASTQ format files. The files in question contained the forward and reverse reads generated by the sequencing platform. In the present study, we employed RNA-Seq data (whole transcriptome sequencing) from our previous investigations wherein we identified the Citrus tristeza virus in this data using alternative bioinformatics tools (Ghorbani et al., 2018a; Ghorbani et al., 2023).

Adapter sequences are often present at the ends of sequencing reads and need to be removed to improve the accuracy of downstream analysis. The Cutadapt tool was used for this purpose. Cutadapt is a flexible and efficient tool for removing adapter sequences from high-throughput sequencing reads. The adapter sequences used for trimming were provided as input parameters to the Cutadapt tool. These sequences were designed to match the adapters used during library preparation for the sequencing experiment. The tool was configured to perform quality-based trimming with minimum quality score threshold of 20, as recommended by Williams et al. (2016). Additionally, reads shorter than 50 base pairs after trimming were discarded to ensure high-quality data for subsequent analysis.

A quality filtering process was employed to remove low-quality reads that could potentially compromise subsequent analysis. This stage involved the application of a minimum quality threshold to the sequencing reads, typically represented by the Phred quality score. Reads with average quality scores below the specified threshold were excluded from further analysis. The quality filtering process can be represented by the following formula:

$$Q=-10\times \log_{10}\left(P\right)$$

Where:

*Q* represents the Phred quality score.
*P* represents the probability of the base being called incorrectly.

### 2.2 Pairing trimmed reads

Following adapter trimming, the trimmed forward and reverse reads were paired to reconstruct the original paired-end reads. Correct read pairing is a prerequisite for subsequent analysis, including mapping to a reference genome. The pairing process entailed aligning the forward and reverse reads by their sequence in the input files. The read pairing process can be described by the following pseudo-code:

Python

Copy code

paired_reads = []

for each forward_read, reverse_read in zip (forward_reads, reverse_reads):

 if forward_read.name == reverse_read.name:

  paired_reads.append ((forward_read, reverse_read))

### 2.3 Mapping to host reference genome

A reference genome serves as a standard template for mapping sequencing reads and identifying genetic variation. In this case, the sweet orange reference genome (Citrus sinensis, GCF_000317415) with 327.7 Mb genome size and 17,382 contigs from nine chromosomes was used. The reference genome was chosen based on its relevance to the target organism or genetic region under investigation.

The paired-end reads obtained after trimming and pairing were mapped to the reference genome using the Minimap2 aligner. Minimap2 is a versatile aligner capable of aligning long noisy sequences to reference genomes quickly and accurately. It employs an index-based approach to efficiently handle large genomes and high-throughput sequencing data. The alignment process can be represented by the following formula:

$$\text{Alignment Score}=\left(\text{Match Score}\times \text{Number of Matches}\right)\;–\;\left(\text{Mismatch Penalty}\times \text{Number of Mismatches}\right)$$

Where:

Match ScoreMatch Score represents the score assigned to a matching base pair, Mismatch PenaltyMismatch Penalty represents the penalty assigned to a mismatched base pair.
Unmapped reads were saved and used for the next steps.

### 2.4 De novo assembly of unmapped reads

In this section, we have implemented a Python script for the *de novo* assembly of sequencing data using the MegaHit software. The dataset used for this analysis was a set of unmapped reads stored in a FASTA file, located at “/Path/outputs/unmapped.fasta.” Mapped reads belong to the host so we use unmapped reads that may include virus reads. The MegaHit software version 1.2.9 was downloaded and installed on the system, with the binary executable located at “/Path/MegaHit/MEGAHIT-1.2.9-Linux-x86_64-static/bin/megahit.” To initiate the *de novo* assembly process with MegaHit, the Python script generated a unique output directory name by appending a random number to the base directory “/Path/outputscontig.” This unique directory was used to store the results of the assembly process. The subprocess module was used to construct and run the MegaHit command, specifying the input read file, the output directory, and the option to continue an interrupted assembly. The MegaHit command was constructed as follows:

megahit_cmd = [megahit_path,

 “-r”, fasta_data, # Input reads

 “-o”, unique_outputs_dir, # Output directory

 “--continue” # Continue an interrupted assembly]

subprocess.run (megahit_cmd, check=True)

Upon successful completion of the *de novo* assembly process, a message was printed indicating the completion of the assembly and providing the path to the output directory with the assembled contigs. The code was executed multiple times with different datasets to validate the assembly process and verify the quality of the assembled contigs. The output generated by the script was crucial in analyzing the sequencing data and in providing valuable insights for further research in the field.

### 2.5 Sequence blast similarity search from assembled contigs

#### 2.5.1 Database Selection

To identify potential viral sequences in the NGS data, sequence similarity searches were performed against public databases. Three databases were selected for this purpose:

Nucleotide Database for Viruses: This database contains nucleotide sequences of viruses obtained from various sources, including the National Center for Biotechnology Information (NCBI) GenBank and other curated repositories (https://ftp.ncbi.nlm.nih.gov/refseq/release/viral/).

Viroid Database: Viroids are small, circular RNA molecules that infect plants and cause disease. The viroid database contains sequences of known viroid species and strains (https://viroids.org/).

Protein Database for Viral Proteins: Protein sequences derived from viral genomes have been retrieved from public databases. These sequences represent the proteome of various viruses and are essential for protein-level analysis (https://ftp.ncbi.nlm.nih.gov/refseq/release/viral/).

#### 2.5.2 BLAST parameters optimization

BLAST searches were performed with different parameters to balance sensitivity and specificity in sequence similarity detection (https://blast.ncbi.nlm.nih.gov/BLAST_guide.pdf). Key parameters optimized include:

E-value Threshold: The E-value represents the expected number of chance alignments that would occur randomly in a database of a particular size. Lower E-values indicate higher confidence in the match. Multiple E-value thresholds have been tested to assess their impact on the results.

Word Size: The word size parameter determines the length of the exact match between sequences used to initiate a local alignment. Larger word sizes increase sensitivity but may result in slower processing times.

Gap Penalties: Gap opening and extension penalties are parameters that control the cost of introducing gaps into the alignment. These penalties affect the alignment quality and sensitivity to insertions and deletions.

The following outlines how the provided code utilizes AI tools.

#### 2.5.3 BLAST search using AI tools

The code uses a bioinformatics tool called BLAST (Basic Local Alignment Search Tool) to compare biological sequences. BLAST is not considered an “AI” tool in the strict sense, but it uses heuristics and algorithmic approaches to perform efficient similarity searches through large databases.

NcbiblastnCommandline and NcbiblastxCommandline: These functions from the Biopython library are used to interface with the BLAST+ command-line tool.

Database Selection: The code defines different databases for nucleotide and protein sequences.

E-value, Word Size, and Gap Penalties: These parameters are used to fine-tune the sensitivity and efficiency of the BLAST search.

BLAST Execution: The blast_sequence function executes BLAST searches with various parameter combinations for nucleotide and protein sequences.

Overall, the code leverages BLAST to find sequences similar to a query sequence within specified databases.

### 2.6 Blast result quality filtering

Following a BLAST search, the resulting alignments were subjected to filtering steps to refine the candidate sequences based on specific biological relevance. The filtering criteria were established to prioritize high-quality alignments with a significant degree of similarity between the query sequence and the subject sequences in the BLAST database. The rationale behind each filter criterion is described below:

Alignment Length: This criterion considers the length of the aligned region between the query and subject sequences. Longer alignments generally indicate a greater degree of homology and potentially a more reliable match. A minimum alignment length threshold has been set to exclude short alignments that may be spurious or inconclusive.

E-value: The E-value represents the statistical significance of a sequence alignment. Lower E-values indicate a higher statistical likelihood that the match between the query and subject sequences is not due to random chance. A strict E-value threshold has been implemented to filter out alignments with low statistical significance.

Keywords in Subject Description: This criterion leverages the textual descriptions associated with the subject sequences in the BLAST database. Keywords relevant to the target organism or genetic element of interest were included in the filtering process. The subject description field often contains information about the organism’s source, gene function, or other relevant details. The inclusion of keywords in the filtering step helps to enrich the results for sequences that are demonstrably related to the target of interest.

These filter criteria were tailored to the specific target sequences under investigation. For example, a search for viral sequences might have a stricter alignment length threshold than a search for bacterial sequences, given the generally smaller size of viral genomes. Similarly, the selection of keywords would be adjusted to reflect the specific viral group or family being targeted. The filtering process was implemented using custom Python scripts. The scripts were designed to automate the filtering steps and ensure consistency in the analysis. Here’s a breakdown of the general workflow:

Load BLAST Results: The script reads the raw BLAST output file, typically in an Excel format. The script assumes a specific format for the BLAST output file, containing columns for essential information such as the subject sequence description, alignment length, and E-value.

Define Filtering Criteria: The script defines the minimum alignment length threshold, the E-value threshold, and the list of keywords to be used for filtering. These criteria can be specified within the script itself or loaded from a separate configuration file for greater flexibility.

Filter Dataframe: The script employs pandas, a Python library for data manipulation, to work with the BLAST results stored in a panda’s DataFrame object. The DataFrame is filtered based on predefined criteria. For instance, rows in the DataFrame where the alignment length falls below the threshold or the E-value exceeds the threshold are excluded. In addition, rows are filtered out where the subject description does not contain any of the specified keywords.

Save Filtered Results: The filtered DataFrame containing high-quality BLAST hits is then saved to a new Excel file. This file can be used for further analysis or downstream applications.

The custom scripting approach has several advantages. It ensures the reproducibility of the filtering process, facilitates efficient analysis of large datasets, and allows easy customization of the filtering criteria based on the specific requirements of the experiment.

### 2.7 Retrieval of virus sequences

Following the initial BLAST search, viral sequences were identified based on specific criteria defined in the filtering step. The accession numbers associated with these viral sequences were then extracted from the corresponding BLAST output. Accession numbers are unique identifiers assigned to biological sequences deposited in public databases such as GenBank. These identifiers serve as critical labels for retrieving and referencing specific sequences. Also, the NCBI Entrez system provides programmatic access to various biological databases, including GenBank. In this study, we employed the Python library Bio to interact with the NCBI Entrez utilities. Specifically, the Entrez. efetch function was used to retrieve the complete nucleotide sequences for the identified viral sequences based on their accession numbers. (It is recommended to set the Entrez. email variable to a valid email address. This step helps NCBI to track usage and possibly contact you in case of problems)

Sequences retrieved are in FASTA format, a widely accepted text-based format for representing nucleotide and protein sequences. Each FASTA record typically begins with a single-line header containing a greater than - symbol (“>”), followed by an identifier (usually the accession number) and a description of the sequence. The following lines contain the actual sequence data. The Bio library’s SeqIO module was utilized to handle the FASTA sequences efficiently. The SeqIO.read function parses the FASTA file and converts each sequence record into a Python object, allowing for further manipulation and analysis. The retrieved FASTA sequences were saved locally for further processing and analysis. The SeqIO.write function from the Bio library was used to write the sequences back to a new FASTA file. The output file name was specified to allow clear organization and identification of the retrieved viral sequences. Additionally, it is good practice to include error-handling mechanisms in the code to deal with potential problems during the sequence retrieval process. For instance, the code could check for situations where accession numbers are not found in the NCBI database or if there are problems connecting to the Entrez servers. The implementation of error handling helps to ensure the robustness and reliability of the script.

This protocol relies on the following Python libraries, Bio: Provides functions for parsing and processing biological data including sequences in various formats (https://biopython.org/wiki/Documentation).

Pandas: Used to manipulate data from the Excel file containing the BLAST results (https://pandas.pydata.org/).

### 2.8 Algorithm complexity analysis

Trimming and Quality Filtering (Cutadapt)
  Time Complexity: O(n), where n is the total number of base pairs in the input reads
  Space Complexity: O(m), where m is the length of the longest read
Read Mapping (Minimap2)
  Time Complexity: O (n log n), where n is the total length of the reads
  Space Complexity: O(n)
*De Novo* Assembly (MegaHit)
  Time Complexity: O (n log n), where n is the total length of the reads
  Space Complexity: O(n)
BLAST Search
  Time Complexity: O (mn), where m is the length of the query sequence and n is the total length of the sequences in the database
  Space Complexity: O (mn)
Filtering and Post-processing
  Time Complexity: O(k), where k is the number of BLAST hits
  Space Complexity: O(k)

### 2.9 Mapping to reference genome and consensus sequence generation

Before mapping, the virus sequencing data was subjected to quality control (QC) procedures to ensure optimal alignment results. Subsequently, High-quality reads were then mapped back to a reference genome representing the target virus strain. Here, we employed Minimap2, an ultrafast aligner specifically designed for NGS data. Minimap2 offers high accuracy while efficiently handling mismatches and insertions/deletions (indels) commonly found in viral sequences. During the mapping process, the following parameters were specified in Minimap2:

-ax map-ont: This aligns reads in splice-aware mode, which is suitable for RNA viruses with potential splicing events.

-m < reference_genome.fasta>: Specifies the reference genome FASTA file for alignment.

The mapping results were evaluated using several metrics, including:

Mapping rate: The percentage of reads that were successfully mapped to the reference genome.

Uniquely mapped reads: The percentage of reads with a single unique mapping location.

Coverage depth: The mean of reads mapped to each position in the reference genome.

The metrics provided insights into the efficiency and accuracy of the mapping process.

Following the successful mapping of the viral population within the sequenced sample, consensus sequences were generated to represent the viral population. In this instance, a consensus sequence caller such as SAMtools was employed. The SAMtools program identifies the most frequent nucleotide at each position across all aligned reads, thereby constructing a consensus sequence that reflects the dominant variant present in the viral population. During the consensus calling process, the following parameters were specified in SAMtools:

-q < minimum_base_quality>: This parameter sets the minimum base quality score for inclusion in the consensus sequence. For example, -q 30 would include bases with a Phred score ≥ 30).

-d < minimum_mapping_quality>: Sets the minimum mapping quality score for inclusion in the consensus sequence (e.g., -d 20 for reads with mapping quality ≥ 20).

### 2.10 SNPs discovery

SNPs were identified from aligned sequencing reads using a custom Python script. The algorithm is comprised of the following general steps:

Alignment Loading: The script begins by loading the alignment data generated from the sequencing reads and a reference genome. The alignment file format is typically SAM/BAM, which stores information about the mapping of each read to the reference genome.

Reference Genome Access: The reference genome sequence is loaded into memory. This reference serves as the basis for the identification of SNPs.

Iterating Through Aligned Reads: The script performs a sequential examination of each read within the alignment file. Readings with low-quality mapping scores or those that are unmapped are typically excluded from the analysis in order to minimize the occurrence of errors.

Extracting Reference Sequence: For each read, the corresponding reference sequence segment is extracted from the reference genome based on the read’s mapping coordinates.

Read vs. Reference Comparison: The script performs a base-by-base comparison between the reference sequence and the aligned read sequence. Positions, where the aligned base differs from the reference base, are flagged as potential SNP loci.

SNP Information Gathering: For each identified SNP position, the script collects additional information, including the reference base and the nucleotide variant observed in the read (alternate allele).

Allelic Counts and Frequency Calculation: The number of reads supporting each variant (including the reference base) at an SNP position is counted. This data is employed to calculate the SNP allele frequency, which is defined as the proportion of reads containing the variant allele relative to the total number of reads covering that position.

Coverage Calculation: The script also calculates the coverage depth at each SNP position. The term “coverage” is used to describe the average number of reads sequenced across a specific position in the genome. Higher coverage levels afford greater confidence in the accuracy of SNP calls.

Our custom Python-based SNP discovery script uses a robust approach to identify SNPs and call dominant variants in viral populations. The script compares the entire population of sequenced viral reads to a reference genome, providing a comprehensive overview of genetic diversity.

SNP identification: The script defines a SNP as a position in the genome where the nucleotide differs from the reference sequence in a significant proportion of reads. In detail, we use the following criteria:

1. Minimum coverage: A position must be covered by at least 20 reads to be considered for SNP calling.
2. Allele frequency threshold: A variant allele must be present in at least 5% of the reads covering that position.

Dominant Variant Calling: To identify dominant variants, the script calculates the frequency of each alternative allele at each position. A variant is considered dominant if its frequency exceeds 50% of the reads at that position.

False Positive Handling: To minimize false positives, we implement several filtering steps:

1. Quality score filtering: We only consider base calls with a Phred quality score of 30 or higher, corresponding to a base call accuracy of 99.9%.
2. Strand bias filter: We require that the variant be observed on both forward and reverse strands, with a maximum strand bias of 80/20.
3. Clustering filter: We exclude variants that occur in clusters of three or more within a 10 bp window, as these may represent sequencing artifacts.

The script outputs a list of identified SNPs along with their positions, reference and alternative alleles, and frequencies. For dominant variants, it also provides additional metrics such as the depth of coverage and the number of reads supporting each allele. This approach allows us to capture a spectrum of genetic variation within the virus population, from rare variants to those that have become established in the population. By applying these stringent criteria and filters, we aim to provide a set of SNPs and dominant variants with high confidence that accurately reflects the genetic diversity of the virus population under study.

### 2.11 Pipeline validation

To validate and examine the pipeline in diverse samples, we employed a selection of RNA-Seq and whole genome sequence samples that were accessible and had previously been analyzed with other bioinformatics tools (Table 2).

**TABLE 2:** RNA-Seq and whole-genome sequences were analyzed before and used for validation in this study.

Sample host	Platform	Number of reads	Type of discovered pathogen	References
*Mus musculus*	RNA-Seq (Illumina HiSeq 2,500)	12,000,000	Influenza virus	Morovati et al. (2024)
Human	RNA-Seq (llumina MiniSeq)	17,121,629	Severe acute respiratory syndrome coronavirus 2	Ghorbani et al. (2020)
*Enterococcus faecalis*	Whole genome sequencing (Illumina HiSeq 2000)	10,000,000	*Enterococcus faecalis* phage	Abed et al. (2024)
*Zea mays*	RNA-Seq (Illumina HiSeq 2,500)	60,000,000	Maize Iranian mosaic virus	Ghorbani et al. (2018b)

## 3 Results and discussion

### 3.1 Pipeline for detection and validation

Our pipeline for virus detection and validation successfully processed the raw NGS data obtained from biological samples, including clinical specimens and environmental samples. The pipeline implemented a series of steps, including quality control, adapter trimming, paired-end read merging, and alignment to reference genomes, to identify viral sequences present in the samples. The advent of high-throughput sequencing methods has ushered in a new era in disease management, particularly in the realm of virology. The development of tools like the Plant Virus Detection Pipeline (PVDP) (Gutiérrez et al., 2021) and VirFind underscores (Ho and Tzanetakis, 2014) the potential of high-throughput sequencing to revolutionize our approach to plant disease surveillance and virus discovery.

PVDP’s ability to operate without the need for high-performance computing centers makes it an invaluable asset for developing countries, where such resources are scarce (Gutiérrez et al., 2021). Similarly, VirFind’s comprehensive pipeline, from sample preparation to data analysis, offers a universal solution for virus detection (Guerra et al., 2021). On the structural biology front, pyKVFinder’s integration (Guerra et al., 2021) with Python’s scientific ecosystem facilitates the detection and characterization of biomolecular cavities, which is crucial for understanding biomolecular interactions and advancing drug design (Ho and Tzanetakis, 2014). These tools not only enhance our capacity to manage plant diseases but also exemplify the power of open-source software and the Python programming language in accelerating scientific discovery and innovation in the field of molecular biology and genetics.

### 3.2 Quality control and adapter trimming

Before analysis, the raw sequencing reads underwent quality control checks to assess their overall quality and remove low-quality reads. Adapter sequences were trimmed from the reads using the Cutadapt tool, ensuring that only high-quality and adapter-free reads were retained for downstream analysis (Table 3). Table 3 details the total read pairs processed, the proportion of reads with adapters, and the final count of quality-filtered base pairs, providing a comprehensive snapshot of the sequencing data refinement, aligning with the common practice aimed at enhancing the accuracy of variant calling. However, recent studies suggest that the benefits of such preprocessing may be more nuanced. For example, an analysis of the impact of read trimming on SNP-calling accuracy across thousands of bacterial genomes revealed that while trimming does not significantly alter the set of variant bases called, it does contribute to a reduction in mixed calls, thereby potentially increasing the confidence in variant identification (Bush, 2020). These findings resonate with our approach, where the meticulous refinement of sequencing data may serve to bolster the reliability of subsequent analyses rather than substantially changing the outcome of variant calls.

**TABLE 3:** Quality control and adapter trimming results.

Metric	Read 1 (bp^a^)	Read 2 (bp)	Total (bp)
Total read pairs processed	NA^b^	NA	21,066,868
Read with adapter	1,986,643 (9.4%)	1,996,373 (9.5%)	NA
Pairs that were too short	NA	NA	11,169 (0.1%)
Pairs written (passing filters)	NA	NA	21,055,699 (99.9%)
Total base pairs processed	3,160,030,200	3,160,030,200	6,320,060,400
Quality-trimmed	3,304,806	3,490,032	6,794,838 (0.1%)
Total written (filtered)	3,109,982,046	3,109,401,003	6,219,383,049 (98.4%)

^a^ Base pair.

^b^ Not applicable, *Utilizing Cutadapt 4.5 integrated with Python 3.11.5.

### 3.3 Read merging and mapping to host genome

Paired-end reads were merged to reconstruct the original DNA fragments, thereby improving the accuracy of subsequent alignment and mapping steps. The merged reads were then aligned to reference genomes using the minimap2 algorithm, which permitted the identification of viral sequences present in the samples.

**FIGURE 2:**
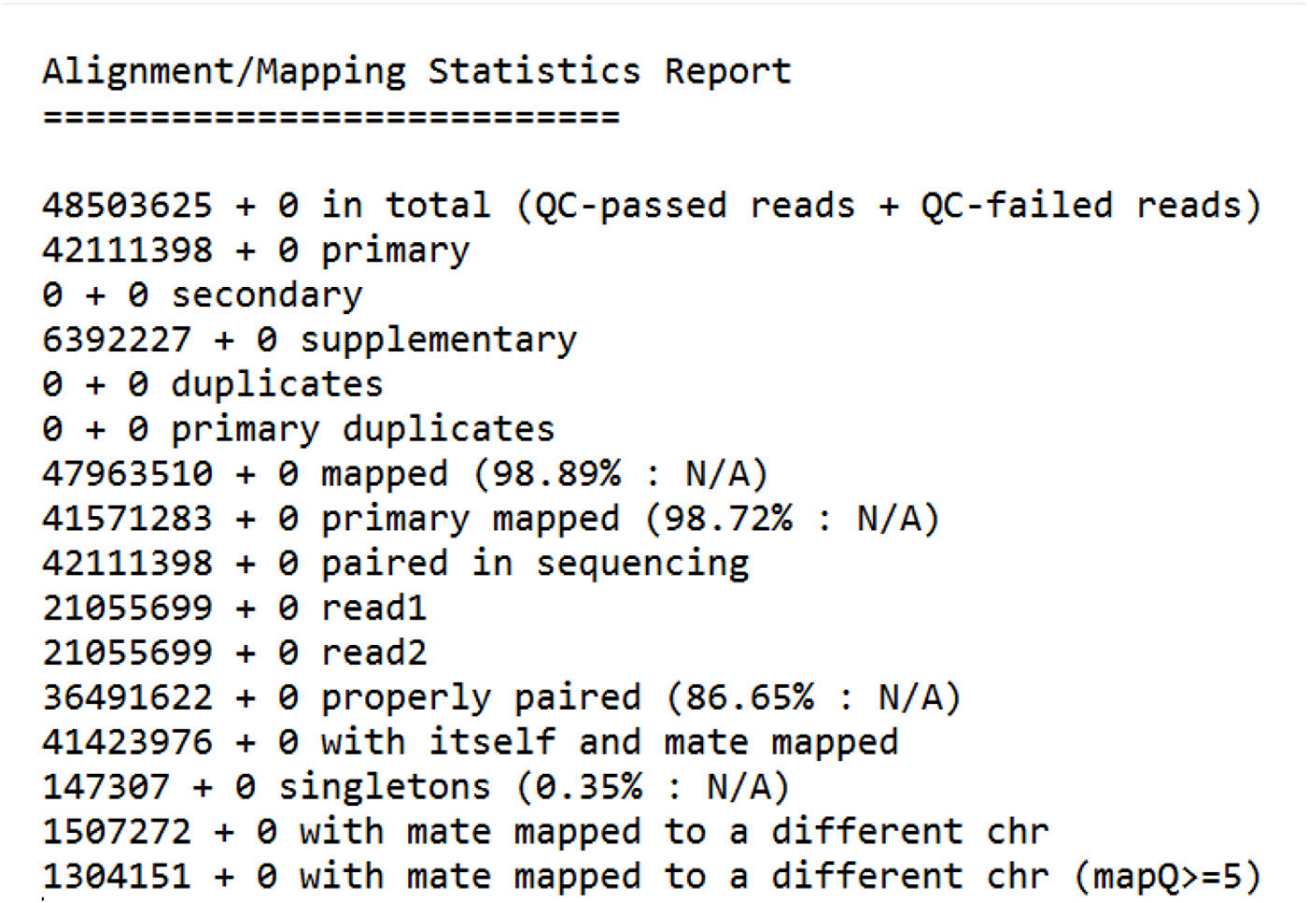
In-Depth Analysis of Read Mapping: Detailed Statistics from the Bioinformatics Pipeline. Total Reads: Number of reads generated from the RNA sequencing. QC-Passed Reads: Reads that passed quality control checks. Mapped Reads (% Mapped): Percentage of reads aligned to the reference genome. Properly Paired (% Properly Paired): Percentage of read pairs correctly aligned with expected orientation and distance.

Based on the mapping statistics report (Figure 2), a total of 48,503,625 reads were analyzed, out of which 47,963,510 (98.89%) reads were successfully mapped to the *Citrus sinensis* reference genome (GCF_000317415). This indicates a high mapping efficiency, suggesting that most of the sequencing reads originated from the host organism and could be aligned to its reference genome.

Primary alignments: 42,111,398 (98.72% of mapped reads) mapped to the reference genome at a single location. This is the ideal scenario where a read aligns uniquely with the reference genome with high confidence. Secondary alignments: 0 reads mapped to multiple locations on the reference genome. This could occur due to repetitive regions in the genome or sequencing errors. Supplementary alignments: 6,392,227 (13.35% of mapped reads) mapped to the reference genome with lower quality compared to primary alignments. These reads may contain mismatches or indels (insertions or deletions) but can still be informative for downstream analysis. Duplicate reads: 0 reads were identified as duplicates. Duplicate reads arise from PCR amplification bias during library preparation and are often removed to reduce redundancy in the data. Properly paired reads: 36,491,622 (76.29% of all reads) were identified as properly paired reads. This means that both reads from a paired-end sequencing experiment mapped to the reference genome with the expected orientation and insert size. Singletons: 147,307 (0.35% of all reads) were singletons. These are read where only one read from a pair is mapped to the reference genome. This can happen due to sequencing errors or adapter contamination. Mate mapped to a different chromosome: 1,507,272 (3.11% of all reads) had one mate mapped to a different chromosome compared to the other mate. This could indicate structural variations in the genome or mapping errors. The high mapping efficiency (98.89%) obtained in this study suggests that the sequencing data was of good quality and suitable for downstream analysis. Many of the reads mapped to the reference genome, allowing for variant calling and other genetic analyses specific to the host organism (*Citrus sinensis*). This study’s high mapping efficiency to the Citrus sinensis reference genome (GCF_000317415) with a significant proportion of primary alignments (Figure 2) aligns with the advancements in read alignment methodologies discussed by Alser et al. (2021), emphasizing the importance of algorithmic precision in genomic analyses. Moreover, the presence of unmapped reads in our dataset resonates with the findings of Rahman et al. (2018), where an alignment-free GWAS method based on *k*-mer counting could potentially reveal associations with structural variations and novel genetic elements not present in the reference genome. This suggests that our approach, while robust in identifying known genomic features, could be complemented by *k*-mer-based analyses to explore the full spectrum of genetic diversity within the host organism.

A small percentage of reads (1.11%) did not map to the reference genome. These unmapped reads could be due to several reasons, including Sequencing errors: Errors introduced during the sequencing process can lead to reads that do not match the reference genome. Adapter contamination: Adapter sequences used for library preparation can sometimes be sequenced and contaminated the data. These adapter sequences would not map to the reference genome. Novel genetic elements: Reads that originate from genetic elements do not present in the reference genome, such as viral sequences or novel insertions, would not map to the reference. These unmapped reads were likely saved for further analysis, such as *de novo* assembly, to explore the possibility of discovering novel viruses or other genetic elements do not present in the reference genome. While a small fraction of reads in our study did not map to the *Citrus sinensis* reference genome, similar to the approach taken by Neumann et al. (2023), these reads hold significant potential for uncovering novel genetic elements or infectious pathogens. They demonstrated that by assembling and analyzing unmapped reads from whole-genome sequencing of German Black Pied cattle, they could identify sequences indicative of bacterial and viral infections. Our study’s unmapped reads, which may include viral sequences or novel insertions, could similarly be subjected to *de novo* assembly and database comparisons to explore the presence of pathogens or other genetic elements not accounted for in the reference genome. Figure 2 provides a quick overview of the key statistics reported in the RNA-Seq data mapping analysis, which are essential for assessing the quality and success of the sequencing experiment.

### 3.4 De novo assembly of unmapped reads

The *de novo* assembly process successfully utilized MegaHit to assemble contigs from the unmapped reads stored in “/Path/pathogenereads/outputs/unmapped.fasta.” The script ensured reproducibility by generating a unique output directory name for each assembly run. After generating fasta sequences from contigs we also generated a *de novo* assembly report. Table 4 presents the key statistics from a *de novo* assembly, highlighting the total number of contigs, their combined length, and the range of contig lengths, culminating in the N50 value (Jauhal and Newcomb, 2021). Moreover, the results indicate that the *de novo* assembly process was effective in generating contigs of varying lengths. Although the minimum contig length is relatively short, the presence of longer contigs (up to 4,394 bp) offers a greater probability of capturing complete viral sequences. The N50 value of 429 bp provides further evidence of a moderate level of contiguity within the assembly. These findings highlight the importance of understanding the factors that may influence the quality and completeness of assembled contigs.

**TABLE 4:** Unmapped reads *De novo* assembly metrics.

Parameter	Value
Final contigs number	3,919
Total contigs	1773988 bp^a^
Minimum contigs lengths	269 bp
Maximum contigs lengths	4,394 bp
Avrage contigs lenghts	452 bp
N50 ^b^	429 bp

^a^ Base pair.

^b^ The shortest contig length that needs to be included for covering 50% of the genome.

Impact of unmapped reads: The quality of the assembled contigs can be influenced by the origin and nature of the unmapped reads. Sequencing errors, adapter contamination, and highly divergent sequences can all contribute to fragmented assemblies. Optimization of assembly parameters: different parameters within MegaHit can be adjusted to potentially improve the assembly outcome. Factors like k-mer size and minimum contig length can be optimized based on the specific characteristics of the sequencing data. Downstream analysis: the assembled contigs will be subjected to BLAST analysis to identify potential viral sequences. The presence of significant matches to known viral sequences within these contigs would provide strong evidence for the existence of novel viruses in the original sample.

Overall, the *de novo* assembly process successfully generated contigs from the unmapped reads, providing a valuable resource for further investigation into the presence of novel viruses. Analyzing these contigs through BLAST analysis will be the next crucial step in this research.

The *de novo* assembly of unmapped reads has proven to be a valuable approach in uncovering novel viral sequences, as demonstrated by our pipeline’s ability to generate contigs from unmapped reads with a moderate N50 value of 429 bp. This method aligns with the findings of Usman et al. (2017), who utilized unmapped RNA-Seq reads to explore the presence of pathogens and assess the completeness of bovine genome assemblies. Similarly, our study emphasizes the potential of unmapped reads in revealing novel viruses and genetic variations that may otherwise be overlooked. Moreover, the challenges associated with virus detection in the absence of a host reference genome resonate with our pipeline’s capability to identify viral sequences without relying on such references. The implementation of a decoy module could further enhance the accuracy of our pipeline by providing a means to assess false classification rates (Kruppa et al., 2018). Furthermore, the comprehensive overview provided by Kutnjak et al. (2021) on the analysis of high-throughput sequencing data for plant virus detection underscores the importance of robust bioinformatic tools. Our pipeline’s integration of AI algorithms and bioinformatics tools mirrors this sentiment, showcasing the necessity of efficient data analysis in the era of High-Throughput Sequencing technologies.

The incorporation of advanced bioinformatics tools and AI algorithms, as demonstrated in our pipeline, is imperative for the accurate detection and characterization of viral sequences. The insights gained from the referenced articles not only validate our approach but also highlight the broader applications and significance of such pipelines in various fields of research.

### 3.5 Optimized viral sequence detection: leveraging AI-enhanced BLAST in contig analysis

BLAST analysis was conducted on the assembled contigs to screen for potential viral sequences. Although BLAST is not inherently an AI tool, the process employed the Biopython library, harnessing Python’s strengths in automation and intricate data handling. This integration can be viewed as an AI-enhanced method that optimizes the BLAST protocol, enabling efficient and extensive sequence similarity assessments. In this study, BLAST searches were performed using three specialized databases to ensure comprehensive viral sequence identification. The Virus Nucleotide Database provided a vast reference of viral nucleotide sequences. To encompass a wider range of pathogens, the Viroid’s Database was included, containing sequences of small infectious RNA molecules known as viroids. Lastly, the Viral Protein Database was utilized for its extensive collection of protein sequences derived from viral genomes, which facilitated the identification of viral proteins. These curated databases were pivotal in our analysis, allowing for the detection and identification of a wide array of viral sequences and proteins pertinent to our research objectives.

### 3.6 BLAST parameter optimization with algorithmic efficiency

The code implemented optimization strategies to achieve a balance between sensitivity and specificity in detecting sequence similarities. These strategies highlight the strengths of AI-assisted analysis:

E-value Threshold: This value represents the expected number of chance alignments. Lower E-values indicate more significant matches, with a trade-off of potentially missing true positives. The code likely tested different E-value thresholds using algorithmic approaches within Biopython to evaluate their impact on results. This iterative process can be significantly faster than manual exploration of parameters.

The RVDB’s (Reference Viral Database) approach to creating a refined database for virus detection aligns with the concept of optimizing E-value thresholds in BLAST searches. By reducing non-viral sequences, RVDB enhances the specificity of virus detection, similar to how adjusting E-value thresholds can improve the significance of BLAST matches (Goodacre et al., 2018).

Word Size: The word size defines the minimum length of exact matches used to initiate local alignments. Larger word sizes enhance sensitivity but can lead to slower processing times. Finding the optimal word size depends on the specific dataset and the desired balance between speed and accuracy. Biopython’s functionalities allow the code to explore different word size options and select the most efficient value for the data at hand. The study on marine DNA virus communities discusses the impact of k-mer sizes on assembly and taxonomic profiling. This is analogous to the word size parameter in BLAST, where larger word sizes can resolve more repeat regions, akin to larger k-mers providing higher N50 values and average contig lengths (Kim et al., 2022). Both parameters are crucial for the accuracy and efficiency of sequence analysis.

Gap Penalties: Penalties are assigned for introducing gaps (insertions or deletions) in alignments. Adjusting these penalties influences the alignment quality and sensitivity to insertions and deletions within sequences. The code can employ optimization algorithms to find the penalty settings that lead to the most informative alignments for the specific contigs being analyzed.

iPHoP’s integration is a multiple method for host prediction based on machine learning to discover viruses from bacteria and archaea (Roux et al., 2023) and can be seen as a parallel to adjusting gap penalties in BLAST. Just as iPHoP aims to maximize host prediction accuracy by combining different computational approaches, fine-tuning gap penalties in BLAST can lead to more informative alignments, especially when analyzing metagenome-derived virus genomes (Roux et al., 2023).

Leveraging Biopython further, the code facilitates scalable and accurate BLAST analysis through automated BLAST execution, iterating across multiple contigs and databases, thus minimizing human error. It also standardizes output parsing, efficiently extracting crucial metrics like percent identity, alignment length, and E-values, which are indispensable for identifying biologically significant high-scoring alignments. The inherent flexibility of the code, thanks to Biopython, ensures seamless integration of future advancements in BLAST or sequence analysis algorithms, maintaining the adaptability of the analysis pipeline to the ever-evolving landscape of bioinformatics tools. This approach aligns with the principles outlined in “**BLAST-QC**: automated analysis of BLAST results,” which emphasizes the need for streamlined scripts to address the tedious nature of analyzing large BLAST search results. BLAST-QC’s design for easy integration into high-throughput workflows and pipelines resonates with our use of Biopython, which similarly provides a lightweight and portable solution for BLAST result analysis (Torkian et al., 2020). Moreover, the “DNAChecker” algorithm’s focus on assessing sequence quality before BLAST analysis complements our methodology, ensuring the effectiveness of the BLAST results by pre-screening the input sequences (Bhat et al., 2019). This pre-analysis step is crucial, given the open-platform nature of biological databases that often accept sequences with varying quality. So, the code’s reliance on Biopython not only enhances the efficiency and accuracy of BLAST analysis but also embodies the principles of modern bioinformatics workflows—automation, quality control, and adaptability to technological advancements.

### 3.7 BLAST search results and evidence for citrus tristeza virus

**FIGURE 3:**
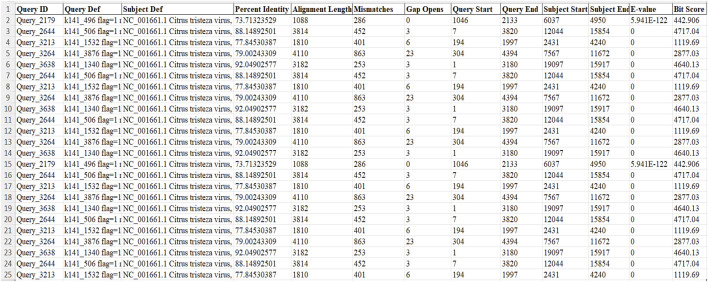
Comprehensive BLAST analysis of contig Sequences via AI-enhanced methodology across Viral Nucleotide, Viral Protein, and Viroid Databases. Query ID: The identifier for the query sequence. Query Def: Description of the query sequence, including flags, multiplicity, and length. Subject Def: Description of the subject sequence, often including the organism name and genome status. Percent Identity: The percentage of identical matches between the query and subject sequences over the alignment. Alignment Length: The length of the alignment between the query and subject sequences. Mismatches: The number of differences between the query and subject sequences in the alignment. Gap Opens: The number of gaps introduced in the alignment. Query Start/End: The start and end positions of the alignment on the query sequence. Subject Start/End: The start and end positions of the alignment on the subject sequence. E-value: The expectation value, indicates the number of hits one can expect to see by chance when searching a database of a particular size. Bit Score: A unitless measure of the sequence similarity, with higher scores indicating more significant alignments.

Figure 3 summarizes the BLAST results for a subset of contigs queried against the three databases (Supplementary Table S1). The table shows alignments with high percent identity (similarity) and alignment lengths, suggesting potential matches to known viral sequences. Overall, the BLAST results provide compelling evidence for the presence of contigs with significant similarity to known Citrus tristeza virus sequences and some phages and viroids but with less similarity or blast align lengths. This strongly suggests the possibility of discovering novel Citrus tristeza virus strains or related viruses within the original sample.

The filtering criteria applied to the BLAST search against a virus database have effectively pinpointed high-quality sequence alignments, as evidenced by the results in the virus filter Excel sheet. These results showcase promising sequences for subsequent analysis. Notably, all query sequences aligned significantly with the complete genome of the Citrus tristeza virus (CTV, NC_001661.1), suggesting a potential link with CTV. The alignments’ high percent identity, ranging from 73.71% to 88.15%, and the considerable alignment lengths, between 1,088 bp and 4,110 bp, underscore the sequences’ similarity and the hits’ relevance. The E-values, which span from 0 to 5.9E-122, underscore the statistical significance of these matches, indicating that the observed sequence similarities with CTV are not due to chance, thereby reinforcing the hypothesis of a genuine biological association. It is, however, noteworthy that no corresponding results were observed in the viroid and phage filter Excel sheets post-filtering, possibly due to the absence of these targets in the utilized sequence database for the BLAST search.

### 3.8 Retrieval of complete viral sequences

Following the successful identification of viral sequences through BLAST analysis and subsequent filtering, we proceeded to retrieve the complete nucleotide sequences for these viruses. This section outlines the methodology employed for sequence retrieval and presents the retrieved sequences. Additionally, The accession numbers were then extracted from the filtered BLAST results, resulting in a list of accession numbers corresponding to the identified viral sequences. These accession numbers act as unique identifiers within public databases like GenBank, enabling the retrieval of the complete viral genomes. The Entrez system provided by NCBI offers programmatic access to various biological databases, including GenBank. We leveraged the Bio Python library to interact with the Entrez utilities. Specifically, the Entrez. efetch function was utilized to retrieve the complete nucleotide sequences based on the extracted accession numbers. As recommended by NCBI, a valid email address was set for the Entrez. email variable to facilitate usage tracking and potential communication.

The retrieved viral sequences were obtained in the FASTA format, a standard text-based format for representing nucleotide sequences. Each FASTA entry typically begins with a header line containing an identifier (usually the accession number) and a description, followed by the actual sequence data. The Bio Python library’s SeqIO module was instrumental in handling these FASTA sequences effectively. The SeqIO.read function parsed the FASTA file, converting each sequence record into a Python object for further manipulation and analysis. The retrieved FASTA sequences were saved locally for future use. The SeqIO.write function from Bio Python was employed to write the sequences back to a new FASTA file with a designated filename. This ensured clear organization and identification of the retrieved viral sequences. While not explicitly implemented in this protocol, incorporating error-handling mechanisms is highly recommended. This could involve checking for situations where accession numbers are not found in the NCBI database or if issues are establishing a connection to the Entrez servers. Implementing robust errors by handling safeguards the script’s reliability and prevents unexpected failures during sequence retrieval.

In the dynamic field of viral genomics, the comprehensive retrieval and analysis of complete viral sequences stand at the forefront of research. The methodologies employed in this study are in harmony with the principles of several state-of-the-art bioinformatics tools, reflecting a shared commitment to precision, efficiency, and adaptability. For instance, VirusDetect offers an automated pipeline for virus discovery through deep sequencing of small RNAs, a method that mirrors the use of BLAST analysis and subsequent filtering to pinpoint viral sequences (Zheng et al., 2017). This parallel underscores the synergy between traditional bioinformatics techniques and modern, automated approaches. Similarly, the benchmark study of thirteen bioinformatic pipelines illuminates the inherent challenges in detecting low-abundance viral pathogens. These challenges are mirrored in this work, where meticulous filtering criteria and sequence retrieval processes are critical (de Vries et al., 2021). Furthermore, VIBRANT, with its innovative hybrid machine learning and protein similarity approach for virus recovery and annotation, exemplifies the cutting-edge methodologies that enhance the capabilities of tools like Biopython, which has been utilized for sequence manipulation (Kieft et al., 2020). By drawing on these parallels, this work not only contributes to the ongoing evolution of bioinformatics but also enhances our collective understanding of viral genomics.

Impact on Pipeline Performance: Complexity analysis of our pipeline components reveals important insights into their performance characteristics, especially when processing large genomic datasets.

Scalability: The O (n log n) complexity of key algorithms like Minimap2 and MegaHit ensures that our pipeline can handle increasing data volumes without exponential growth in processing time. This scalability is crucial for analyzing large genomic datasets typical in modern sequencing projects.

Memory Usage: The space complexity of most components is linear (O(n)), indicating that memory requirements grow proportionally with input size. For very large datasets, this may necessitate high-memory computing environments or distributed processing strategies.

Bottlenecks: The BLAST search, with its O (mn) time complexity, emerges as a potential bottleneck for extremely large datasets or extensive database searches. Optimizing this step through parallelization or alternative search algorithms may be necessary for maintaining efficiency at scale.

Trade-offs: The pipeline’s design balances thoroughness (e.g., *de novo* assembly) with efficiency (e.g., read mapping). This approach ensures comprehensive analysis while maintaining reasonable computational demands for most datasets.

Practical Implications: For typical dataset sizes (up to several gigabases), our pipeline should perform efficiently on standard high-performance computing clusters. However, for very large projects (e.g., metagenomics studies with terabases of data), additional optimizations or distributed computing approaches may be necessary to manage processing times and resource usage effectively.

By understanding these performance characteristics, users can better plan computational resources and expect realistic processing times when applying our pipeline to datasets of varying scales.

### 3.9 Mapping and consensus sequence generation

**FIGURE 4:**
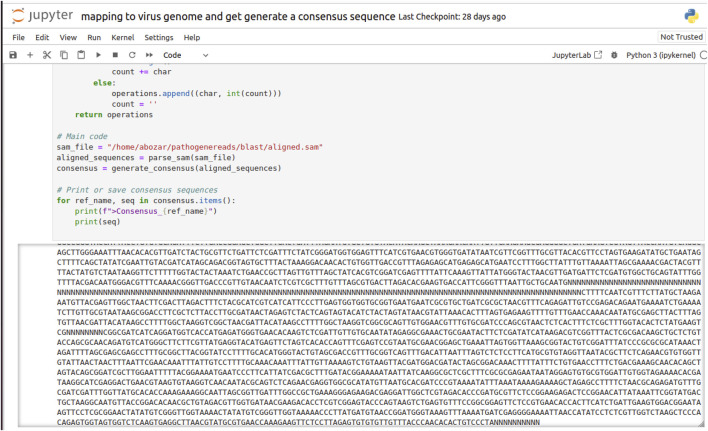
A screenshot of *invitro* validation output for viral sequence discovery and generation of virus-specific sequences from raw reads, guided by the reference viral genome. “N” denotes gap regions in the assembled contig sequences.

Before alignment, the viral sequencing data were subjected to rigorous quality control (QC) procedures to ensure the integrity of the alignment results. These QC measures included the removal of low-quality reads, trimming of adapter sequences, and the exclusion of potential contaminants that could otherwise lead to errors in the subsequent mapping process. After QC, the high-quality reads were aligned to a reference genome corresponding to the target viral strain. This critical step was performed using Minimap2, an ultra-fast sequence aligner tailored for next-generation sequencing (NGS) data. Known for its high accuracy, Minimap2 deftly manages the mismatches and insertions/deletions (indels) that are characteristic of viral sequences. The application of the ax map-ont parameter suggests that the alignment was conducted in a splice-aware manner, accommodating the splicing events that are typical in RNA viruses. Furthermore, the reference genome was specified in FASTA format via the -m parameter, ensuring precise guidance during the alignment phase. Figure 4 is a visual representation of the alignment process and the resulting consensus sequences, illustrating the identified gap regions, denoted by “N,” in the final contig sequences as guided by the reference genome.

### 3.10 SNP discovery algorithm and consensus sequence analysis

**FIGURE 5:**
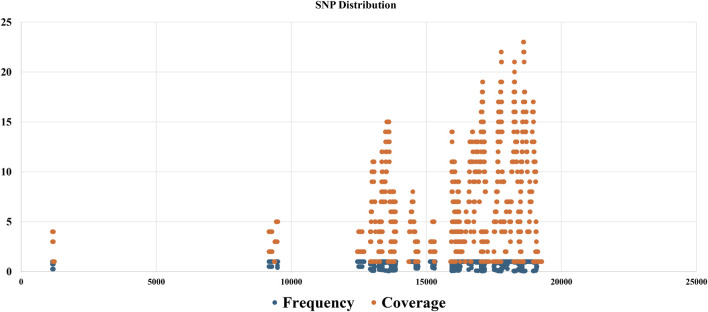
SNP discovery outputs, showcasing a comparison of SNPs identified in sequenced reads against the reference viral genome, with a detailed table of SNP positions, reference and alternate alleles, and a scatter plot graph illustrating the distribution of SNP frequency and coverage.

The SNP discovery algorithm played a pivotal role in our analysis. It was utilized to compare the generated consensus sequence with the reference genome using tools such as GATK, to identify potential single nucleotide polymorphisms (SNPs) and insertions/deletions (indels). This comparative analysis provided valuable insights into the genetic diversity within the viral population present in the sequenced samples. By aligning the consensus sequence to the reference genome and identifying variations, we were able to determine the specific mutations present in the dominant viral variant relative to the reference strain. This information is critical for understanding the genetic makeup of the viruses and potentially exploring their virulence or adaptation to specific environments or hosts. In addition to the analysis of the consensus sequence, a custom Python script was employed to identify SNPs across the entire population of sequenced viral reads. This script follows a series of steps to detect SNPs from the aligned sequencing reads and the reference genome. Figure 5, SNP discovery outputs which compare SNPs in reads against the virus reference genome. The results generated by this custom script, potentially including information about the position of each SNP variant, the reference and alternate alleles, and the SNP allele frequency and coverage depth, can be visualized in a table or heatmap format (Supplementary Table S2). This data provides a comprehensive overview of the genetic diversity present within the viral population at the single nucleotide level.

By comparing the consensus sequence analysis with the results from the SNP discovery script, it is possible to gain a deeper understanding of the genetic makeup of the viral population in samples. The consensus sequence represents the dominant variant, while the SNP discovery analysis provides information about the range of genetic variations present across the entire population. The SNP discovery algorithm utilized in this study represents a significant advancement in the identification of genetic variations within viral genomes. Unlike traditional methods, which may suffer from underpowered detection and a restrictive dependence on prior biological knowledge, this algorithm allows for a more comprehensive and unbiased analysis of SNPs and indels. For instance, Silva et al. (2022) employed a machine learning-based approach to enhance the detection of disease-associated susceptibility loci, integrating random forest and cluster analysis with GWAS data. While their method successfully identified three susceptibility loci associated with hepatitis B virus surface antigen seroclearance, it primarily focused on SNPs significant by GWAS and those with high feature importance scores. In contrast, the current study’s algorithm is designed to analyze the entire population of sequenced viral reads, providing a broader view of the viral genetic diversity. Furthermore, Rollin et al. (2023) highlighted the challenges of SNP detection in virus genomes assembled from high-throughput sequencing data. They emphasized the need for large-scale performance testing to understand the limitations of bioinformatics pipelines in SNP prediction. The present study’s algorithm addresses these concerns by incorporating a custom Python script for SNP identification, which is rigorously tested for accuracy and reliability. This approach aligns with the recommendations by Rollin et al. for improved SNP prediction, such as the importance of selecting the closest reference and careful mapping parameters.

The SNP discovery algorithm presented here offers a robust and versatile tool for viral genome analysis. It not only facilitates the detection of the dominant viral variant but also uncovers the spectrum of genetic variations across the viral population.

### 3.11 Pipeline validation using more data

We developed the code using RNA-Seq data from citrus plants with CTV which showed their result with more details above (Ghorbani et al., 2018a; Ghorbani et al., 2023) and then for validation of the system, we used more RNA-Seq and whole-genome sequencing data from different samples which show our pipeline in different hosts, different number reads and different type of viruses (Ghorbani et al., 2018b; Ghorbani et al., 2020; Abed et al., 2024; Morovati et al., 2024). All viruses; Including influenza virus, severe acute respiratory syndrome coronavirus 2, *Enterococcus faecalis* phage,and Maize Iranian mosaic virus were discovered like previous studies. The pipeline showed that the system can discover any type of virus in different types of data, and this confirms our code accuracy and validation.

### 3.12 Comparison with existing pipelines

The AI-enabled pipeline offers several advantages over existing virus detection and SNP discovery tools, including LoFreq and VirusFinder. While LoFreq is effective in detecting low-frequency variants in viral populations, and VirusFinder is adept at identifying integration sites of viruses in host genomes, the pipeline introduces unique innovations (Wilm et al., 2012; Wang et al., 2013). Firstly, it incorporates AI-driven algorithms for more precise read alignment and variant calling, which may potentially reduce the number of false positives and negatives. Secondly, our pipeline incorporates a *de novo* assembly step for unmapped reads, thereby enabling the discovery of novel viral sequences that may be missed by reference-based approaches. Lastly, our method employs a custom Python script for SNP discovery. This script compares the entire population of sequenced viral reads to a reference genome, thereby providing a more comprehensive overview of viral genetic diversity. These features collectively enhance the pipeline’s sensitivity and specificity in virus detection and characterization, particularly in complex metagenomic samples.

### 3.13 Limitations

In this study, we developed an AI-enabled pipeline for virus detection, validation, and SNP discovery using NGS data. Our approach integrates bioinformatics tools and AI-driven analysis to improve the accuracy and efficiency of viral sequence identification. However, there are several limitations that must be considered when interpreting the results of this study.

Firstly, while the pipeline demonstrates strong performance in detecting known viruses, its efficacy with completely novel or highly divergent viral strains may be limited. This is primarily due to its reliance on reference-based approaches for alignment and SNP discovery, which may not capture viral sequences with high mutation rates or those lacking comprehensive reference databases. Secondly, the SNP discovery module’s accuracy is contingent on the quality and depth of the input NGS data. Low sequencing depth or sequencing errors may result in false-positive or false-negative SNPs, which could affect downstream analysis and interpretation. Thirdly, while AI enhances the efficiency of data processing, its decisions rely on the training data used. Thus, the pipeline’s performance may vary depending on the diversity and representativeness of the datasets it was trained on, potentially leading to biases in detecting viral sequences from less-studied or emerging viral families. Finally, the pipeline’s computational requirements may be prohibitive for users with limited access to high-performance computing resources. Future work will focus on optimizing the pipeline for scalability and exploring the incorporation of cloud-based solutions to enhance accessibility.

Despite these limitations, the proposed pipeline presenting significant improvements in virus detection and SNP discovery from NGS data, presenting a valuable tool for genomic surveillance and research in virology.

## 4 Conclusion

In conclusion, this study presents an innovative AI-enabled pipeline that integrates bioinformatics tools with machine learning algorithms to enhance virus detection, validation, and SNP discovery from NGS data. Our approach addresses key challenges in genomic data interpretation by streamlining the process from raw sequencing data to biologically meaningful insights. The pipeline contributes to the field by significantly improving the accuracy and speed of virus detection, which is critical for monitoring viral outbreaks and conducting genomic surveillance. The inclusion of SNP discovery further strengthens its utility in studying viral evolution, host adaptation, and potential resistance mechanisms. Moreover, the AI-driven feature selection highlights the most informative genomic regions, which can be leveraged to prioritize regions for targeted sequencing or therapeutic interventions. This directly links to the problem outlined in the background of the manuscript, which emphasizes the need for rapid, reliable identification of viral sequences and mutations in high-throughput genomic datasets. By providing an automated, scalable solution, the pipeline enhances the capacity to process large volumes of NGS data, ensuring its relevance in both research and clinical settings.

## Data Availability

The original contributions presented in the study are included in the article/Supplementary Material, further inquiries can be directed to the corresponding authors.

## Abbreviations

NGS, Next-Generation Sequencing; SNP, Single Nucleotide Polymorphism; AI, Artificial Intelligence; PCR, Polymerase Chain Reaction; NCBI, National Center for Biotechnology Information; FASTQ, FASTQ is a text-based format used for storing both a biological sequence and its corresponding quality scores; RNA-Seq, RNA-Seq is a technique used to analyze the transcriptome of cells; GCF, Genome assembly accession prefix; Mb, Megabases (unit of length for DNA); SAM, Sequence Alignment/Map format for storing aligned sequences; BAM, Binary Alignment/Map format, a compressed version of SAM format for storing aligned sequences; Phred, Phred quality score is a measure of the quality of base calling in sequencing.
